# Psychedelic-Assisted Psychotherapy After COVID-19: The Therapeutic Uses of Psilocybin and MDMA for Pandemic-Related Mental Health Problems

**DOI:** 10.3389/fpsyt.2021.716593

**Published:** 2021-09-06

**Authors:** Elena Argento, Devon Christie, Lindsay Mackay, Cody Callon, Zach Walsh

**Affiliations:** ^1^Department of Medicine, University of British Columbia, Vancouver, BC, Canada; ^2^British Columbia Centre on Substance Use, Vancouver, BC, Canada; ^3^Department of Psychology, University of British Columbia, Kelowna, BC, Canada

**Keywords:** psychedelics, mental health, trauma, COVID-19 pandemic, psychedelic-assisted psychotherapy

## COVID-19-Related Impacts on Mental Health

The COVID-19 pandemic stands to have impacts on mental health and well-being that will extend beyond its formal resolution. Before COVID-19, mental health disorders were already challenging global healthcare systems, directly accounting for 7.4% of the total burden of disease (1, 2). An estimated 1 billion people worldwide suffer from a mental health disorder, with the two most common disorders—depression and anxiety—costing the global economy US$1 trillion per year (3). Stigma and limited treatment options have amounted to substantial unmet need and violations in human rights for people with mental health disorders (1, 4, 5). Looking ahead, heightened post-pandemic demand for mental healthcare signifies an urgent need to bolster clinical capacity by integrating novel, cost-effective approaches into existing systems (6).

Emergent literature globally describes the diverse impacts of COVID-19 on mental health (7, 8). For instance, available data among hospitalized COVID-19 patients in China revealed that approximately 96% suffered post-traumatic stress symptoms (9). Studies among intensive care unit (ICU) patients with previous coronaviruses infer high rates of posttraumatic stress disorder (PTSD), depression and anxiety (30-40%) persisting months after discharge (10), with similar rates observed in patients infected with COVID-19 (11). Highly exposed individuals such as frontline healthcare workers are susceptible to similarly negative outcomes due to prolonged occupational stress, elevating risk of PTSD and suicidality (12–14). Importantly, post-pandemic mental disorders are not limited to individuals directly exposed to COVID-19. Rather, research documents PTSD symptoms among individuals who have been indirectly exposed by witnessing (e.g., via the media) or being confronted with the threat of death or serious illness (e.g., worry/anticipation about the future) (7).

COVID-19 has significantly altered lives in ways that exacerbate drivers of mental health problems, with widespread uncertainty, increased experiences of grief and loss, social isolation, economic and housing instability, and decreased access to critical services related to lockdowns (6, 15). Further, available data on the impacts of COVID-19 on substance use patterns indicate increased use of alcohol and other substances in response to stress and negative emotions (8, 16, 17). Social connections are crucial for people struggling with addiction and comorbidities such as depression, and increased social disconnection represents a key risk factor for adverse outcomes (e.g., relapse and overdose) (1, 6, 18). The societal and economic consequences are tremendous, with structurally vulnerable groups at greatest risk of harms. For example, North America has seen dramatic spikes in fatal overdoses attributable to socio-structural conditions worsened by COVID-19 (18, 19), disproportionately impacting racialized groups (20).

The legacy of mental health problems that will be left behind by COVID-19 incites innovative solutions to address rising rates of PTSD, depression, anxiety, addictions, and social disconnection. As such, we would be remiss not to consider a novel approach with anti-depressive, anxiolytic, and antiaddictive potential that may also foster a sense of social and environmental connectedness, known as psychedelic-assisted psychotherapy (21–24).

## Opening Regulatory Doors to Psychedelics

A considerable and growing body of evidence speaks to the potential of psychedelic-assisted psychotherapies to enhance treatments for PTSD, depression, end-of-life anxiety, and substance use disorders (23, 24). The ensuing government, industry and social support includes the US Food and Drug Administration (FDA) granting breakthrough therapy designation for psilocybin and 3,4-methylenedioxymethamphetamine (MDMA) for treatment-resistant depression and PTSD, respectively (24).

A range of jurisdictions worldwide are expanding access to psychedelic-assisted psychotherapies, including through compassionate use or “right-to-try” pathways. In 2019, the Israeli government approved its first Compassionate Use Program for MDMA-assisted psychotherapy, shortly followed by FDA approval for an Expanded Access program in the US (25). Switzerland has permitted compassionate use of MDMA and lysergic acid diethylamide (LSD) since 2014 (26). The state of Oregon has now legalized psilocybin-assisted psychotherapy and decriminalized all drugs, alongside dozens of similar legislative reforms to legalize or decriminalize psychedelic plants and fungi across the US, including bills to expand “right-to-try” laws for people with serious or life-threatening illnesses (27). In Canada, a growing number of permissions have been granted by the federal government to use psilocybin for existential distress, and for therapist training purposes (28). Health Canada recently announced a notice of intent to restore access to psilocybin and MDMA through the Special Access Programme, which followed a national petition signed by nearly 15,000 Canadians in support of decriminalizing psychedelic plants and fungi (29).

Mounting public interest in psychedelic-assisted psychotherapy is reflected in the 2020 Global Drug Survey. Nearly 6% of 110,000 respondents used psychedelics in the past year for self-treatment of mental health conditions, and 90% who had a supervised psychedelic experience in an uncontrolled setting indicated interest in taking psychedelics under a legally regulated and approved treatment system (30). These findings underscore the need for increased public education, training of qualified care providers, and harm reduction approaches as regulatory frameworks evolve.

While, the pandemic has illuminated deficiencies and inequalities in our healthcare systems, it also provides a rare opportunity for change. The collective experience of COVID-19 has rearranged priorities by bringing mortality, loss, and mental health to the forefront, and is generating innovation. Rapidly evolving regulations around psychedelics come at an opportune time and may open doors to new treatment modalities. In this context, below we provide an overview of the clinical evidence for psilocybin- and MDMA-assisted psychotherapy, which, if larger studies continue to validate findings, are poised to make a substantial impact on mental healthcare and bolstering treatments for post-pandemic stress and trauma disorders.

## Psilocybin-Assisted Psychotherapy for Depression and Existential Distress

Clinical evidence for psilocybin-assisted psychotherapy in promoting long-lasting relief from existential distress is highly relevant in a post-pandemic world. Randomized clinical trials (RCTs) have observed large effect sizes sustained 4.5 years after a single dose of psilocybin delivered in the context of psychotherapy: 60–80% of participants with cancer-related existential distress had clinically significant reductions in depression and anxiety (31). Between 1960 and 2017, 11 psychedelic clinical trials involving 445 participants with life-threatening diseases demonstrated significant reductions in symptoms of depression and anxiety, highlighting overall safety and efficacy (22, 32).

Likewise, open-label trials of psilocybin-assisted psychotherapy for treatment-resistant depression have observed rapid and sustained improvements up to 6 months follow-up (33, 34). A recent RCT found psilocybin-assisted psychotherapy produced marked antidepressant effects in patients with major depressive disorder: 71% had clinically significant reductions and 54% achieved remission 4 weeks later, demonstrating effect sizes greater than are typical for psychotherapy alone and for other pharmacological treatments (35). Most recently, a double-blind RCT published during the pandemic that compared psilocybin with a selective serotonin reuptake inhibitor (SSRI), found similar efficacy between the two groups with some secondary outcomes favoring psilocybin (36).

As the pandemic continues to intensify experiences of disconnection and existential distress, it is noteworthy that spiritual or mystical experiences occasioned by psychedelics are thought to mediate several therapeutic outcomes (37–41) by eliciting powerful emotional responses including awe, ego-dissolution, sense of unity, sacredness, and insight (42, 43). Approximately, 70–90% of participants across clinical trials rated their psilocybin experience among the top five most personally meaningful and spiritually significant experiences of their entire lives (38, 39, 44).

## Psilocybin-Assisted Psychotherapy for Substance Use Disorders

Increases in problematic substance use represent an imminent health concern that is likely to linger well beyond the formal conclusion of the pandemic. Given the ongoing overdose crisis and mounting concerns around post-pandemic stress and increasing use of alcohol and tobacco (17, 45, 46), relevant preliminary studies of psilocybin-assisted psychotherapy signal an important area for further investigation. For example, a small, open-label pilot study for tobacco smoking cessation demonstrated high abstinence rates at 6 months and 1 year following two or three doses of psilocybin in combination with Cognitive Behavioral Therapy among long-term cigarette smokers (47, 48). Promising results were also observed in a pilot study for alcohol use disorder, with significant increases in abstinence rates and reduced craving following one or two psilocybin sessions in addition to weekly Motivational Enhancement Therapy (49). These findings have encouraged further development of clinical trials to assess psilocybin-assisted psychotherapy for other substance use disorders.

## MDMA-Assisted Psychotherapy for Trauma-Related Disorders

Several decades of clinical trials have demonstrated the safety and efficacy of MDMA-assisted psychotherapy, culminating in recent findings from a double-blind RCT published in *Nature Medicine*. The phase 3 study found that 67% of participants no longer met clinical criteria for PTSD 2 months following MDMA-assisted psychotherapy, demonstrating rapid treatment efficacy for participants with severe PTSD and comorbidities such as depression, historical childhood trauma and substance use disorders (50). Six prior phase 2 RCTs signaled the promise of MDMA to bolster outcomes of traditional psychotherapy (51), and long-term follow-up demonstrated lasting therapeutic benefits (52). Research remains ongoing in over 16 international jurisdictions with active phase 3 RCTs in Canada, the US and Israel (53). While two SSRIs have been approved for PTSD, pooled data indicate that MDMA-assisted psychotherapy may constitute “a substantial improvement over available pharmacotherapies in terms of safety and efficacy” (54). Other research suggests MDMA-assisted psychotherapy may be efficacious for treating social anxiety among adults with autism, end-of-life anxiety/psychological distress, alcohol use disorder and eating disorders (55–58). Based on these findings, researchers recommend that MDMA-assisted psychotherapy be expeditiously evaluated for clinical use (50). MDMA represents a potentially cost-effective (59) and promising novel enhancement to psychotherapy for trauma-related disorders further challenged in post-pandemic times.

Overall, psychedelic-assisted psychotherapies may have potential to specifically and uniquely address post-pandemic issues, such as PTSD, bereavement, and depression due to their distinct mechanisms of action that are proposed to engage processes related to meaning in life, acceptance of change, and the reiteration of core personal and spiritual values (60, 61).

## Context and Safety

Clinical trials suggest psychedelics are physiologically and psychologically safe (24, 32, 51, 53, 62) when delivered in supportive settings that adhere to guidelines covering a range of factors (63). Psilocybin mushrooms, as with other psychedelic plants, have been used for centuries, if not millennia, as traditional medicines for a wide range of healing purposes without reports of safety concerns (64). In the early era (1977–1985) of clinical study when MDMA was still legal, approximately 4,000 psychiatrists and psychologists administered MDMA-assisted psychotherapy to an estimated 500,000 patients without evidence of serious harm (65, 66). Nevertheless, psychedelic-assisted psychotherapy is not without risk. As regulations trend toward expanding access to psychedelics, thoughtful consideration of “set and setting” (mindset of the patient and the broader physical environment) is needed to ensure safe and adequately supported use of these medicines (63), in addition to expanding therapist training programs (67).

## A Novel Enhancement to Post-Pandemic Mental Healthcare

Amid escalating need for enhanced mental health services in the wake of COVID-19, psychedelic-assisted psychotherapy represents a promising breakthrough treatment, including for refractory conditions (68). Novel alternatives are urgently needed to address the limitations of existing treatment options. Widely prescribed psychiatric medications are ineffective for many who have access, and myriad unwanted side effects are associated with low compliance (69, 70). Further, an estimated 90,000 visits are made annually to US emergency departments due to adverse events (71). Importantly, the novel therapeutic value of psychedelics stems from their role as enhancements to a psychotherapeutic change process grounded in a relationship-centered approach that views mental health through a biopsychosocial lens (68). Psychedelic-assisted psychotherapy holds potential to empower patients by enhancing self-reflection and self-directed change, which may help to facilitate meaningful and lasting benefits to well-being. Given the tremendous economic burden of mental health disorders globally (3, 72), should the benefits described in pilot studies hold consistent in larger RCTs, there are considerable potential cost savings to healthcare systems.

## Conclusion

Rising rates of pandemic-related stress and trauma disorders call for concerted efforts to find innovative strategies to bolster treatments. Psychedelic-assisted psychotherapy is certainly no quick fix. However, the therapeutic use of psychedelics such as psilocybin and MDMA, carefully and thoughtfully integrated into existing evidence-based interventions denotes one of the most promising paths forward (73). The COVID-19 pandemic underscores longstanding challenges to mental health, but also presents an opportunity to develop promising new therapeutic approaches founded on evidence, compassion, and meaningful connection to bring about lasting benefits to health and well-being.

## Author Contributions

EA conceptualized the topic of the paper with inputs from DC and ZW and wrote the first draft. EA, DC, LM, CC, and ZW all made significant contributions to the manuscript. All authors critically revised and approved the final draft.

## Conflict of Interest

EA, DC, LM, and CC work part-time with Numinus Wellness, a Canadian mental health company interested in the use of psychedelics for medical purposes. Numinus Wellness was not involved in the writing of this manuscript or the decision to submit for publication. ZW is in paid advisory relationships Numinus Wellness, EntheoTech & Synthesis Health, and is an unpaid advisor to the Multidisciplinary Association for Psychedelic Science - Canada and Mycomedica Life Sciences regarding the medical development of psychedelics or related compounds. None of these organizations were involved in the writing of this manuscript or the decision to submit for publication.

## Publisher's Note

All claims expressed in this article are solely those of the authors and do not necessarily represent those of their affiliated organizations, or those of the publisher, the editors and the reviewers. Any product that may be evaluated in this article, or claim that may be made by its manufacturer, is not guaranteed or endorsed by the publisher.
